# Risk Factors for Symptomatic Gallstone Disease and Gallstone Formation After Bariatric Surgery

**DOI:** 10.1007/s11695-022-05947-8

**Published:** 2022-02-10

**Authors:** Sylke Haal, Maimoena S. S. Guman, Sjoerd Bruin, Ruben Schouten, Ruben N. van Veen, Paul Fockens, Marcel G. W. Dijkgraaf, Barbara A. Hutten, Victor E. A. Gerdes, Rogier P. Voermans

**Affiliations:** 1 Department of Internal Medicine, Spaarne Gasthuis, 2134 TM Hoofddorp, the Netherlands; 2 Department of Gastroenterology and Hepatology, Amsterdam Gastroenterology Endocrinology Metabolism, Amsterdam UMC, University of Amsterdam, 1105 AZ Amsterdam, the Netherlands; 3 Department of Internal and Vascular Medicine, Amsterdam Gastroenterology Endocrinology Metabolism, Amsterdam UMC, University of Amsterdam, 1105 AZ Amsterdam, the Netherlands; 4 Department of Surgery, Spaarne Gasthuis, 2134 TM Hoofddorp, the Netherlands; 5 Department of Surgery, Flevohospital, 1315 RA Almere, the Netherlands; 6 Department of Surgery, OLVG, 1061 AE Amsterdam, the Netherlands; 7 Department of Epidemiology and Data Science, Amsterdam UMC, University of Amsterdam, 1105 AZ Amsterdam, the Netherlands; 8 Department of Epidemiology and Data Science, Amsterdam UMC, University of Amsterdam, Amsterdam Cardiovascular Sciences, 1105 AZ Amsterdam, the Netherlands

**Keywords:** Gallstone formation, Symptomatic gallstone disease, Risk factors

## Abstract

**Purpose:**

Patients who undergo bariatric surgery are at risk for developing cholesterol gallstones. We aimed to identify risk factors that are associated with symptomatic gallstone disease and gallstone formation after bariatric surgery.

**Materials and Methods:**

We included participants of the UPGRADE trial, a multicenter randomized placebo-controlled trial on the prevention of symptomatic gallstone disease with ursodeoxycholic acid (UDCA) after bariatric surgery. The association between patient characteristics and symptomatic gallstone disease, and gallstone formation was evaluated using logistic regression analysis.

**Results:**

Of 959 patients, 78 (8%) developed symptomatic gallstone disease within 24 months. Risk factors were the presence of a pain syndrome (OR 2.07; 95% CI 1.03 to 4.17) and asymptomatic gallstones before surgery (OR 3.15; 95% CI 1.87 to 5.33). Advanced age (OR 0.95; 95% CI 0.93 to 0.97) was protective, and UDCA prophylaxis did not reach statistical significance (OR 0.64; 95% CI 0.39 to 1.03). No risk factors were identified for gallstone formation, whereas advanced age (OR 0.98; 95% CI 0.96 to 1.00), statin use (OR 0.42; 95% CI 0.20 to 0.90), and UDCA prophylaxis (OR 0.47; 95% CI 0.30 to 0.73) all reduced the risk.

**Conclusion:**

Young patients with a preoperative pain syndrome and/or asymptomatic gallstones before bariatric surgery are at increased risk for symptomatic gallstone disease after surgery. Whether statins, either alone or in combination with UDCA prophylaxis, can further reduce the burden of gallstones after bariatric surgery should be investigated prospectively.

**Graphical abstract:**

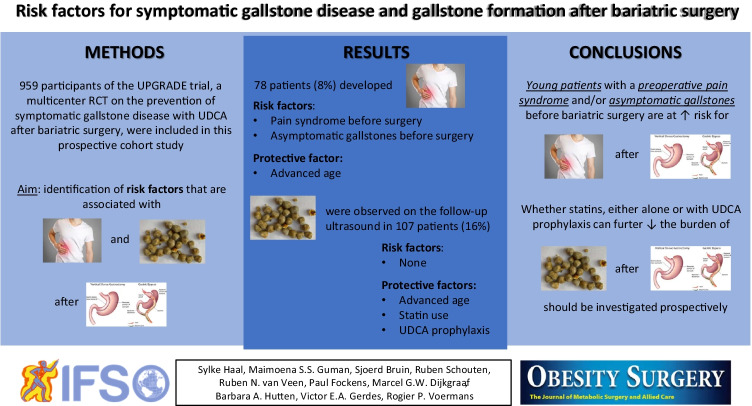

**Supplementary Information:**

The online version contains supplementary material available at 10.1007/s11695-022-05947-8.

## Introduction

Rapid weight loss after bariatric surgery is a major risk factor for cholesterol gallstone disease [1]. Among patients who undergo bariatric surgery, up to 40% develop gallstones [2–4], and approximately 8% to 15% become symptomatic requiring a cholecystectomy [5–7]. Despite extensive research, it is currently not possible to predict the risk for gallstones in the individual bariatric patient. Most previous studies did not identify any preoperative risk factors [2, 3, 8–22], whereas others found inconsistent associations between gender, age, ethnicity, the presence of comorbidities (such as pain syndrome, hypertension, type 2 diabetes, dyslipidemia, fatty liver disease and insulin resistance), the use of statins, or type of surgery on the one hand, and symptomatic gallstone disease or gallstone formation after bariatric surgery on the other hand [4, 23–39]. Although the postoperative weight loss predisposes bariatric patients to develop gallstones, it cannot be predicted beforehand. Furthermore, it remains uncertain whether the variation in speed and amount of the weight loss are responsible for why some patients form gallstones extremely rapid after bariatric surgery (within in 6 weeks), while others do not.

The purpose of this study was to investigate associations between patient characteristics and symptomatic gallstone disease, and gallstone formation after bariatric surgery using data from the UPGRADE trial, a large randomized placebo-controlled trial [40, 41].

## Methods

### Study Design and Patients

The source population for this prospective cohort study comprised the participants of the UPGRADE trial (Netherlands Trial Register, NL5954). The protocol and results of this trial have been described elsewhere [40, 41]. In summary, the UPGRADE trial was a multicenter, randomized, placebo-controlled, double-blind superiority trial conducted in the Netherlands between January 2017 and November 2020. Patients with morbid obesity, an intact gallbladder, and scheduled for laparoscopic Roux-en-Y gastric bypass (RYGB) or sleeve gastrectomy were included. Key exclusion criteria were the presence of symptomatic gallstone disease before bariatric surgery, prior bariatric surgery, and prior gallbladder surgery. Additional exclusion criteria are listed in the trial protocol [40]. All patients received a preoperative ultrasound before randomization to determine the presence of asymptomatic gallstones. The result of the ultrasound was blinded for the patients and treating physicians. Patients were randomly assigned to receive either 900 mg ursodeoxycholic acid (UDCA) daily for 6 months or placebo, and were followed for 24 months. The gallbladder ultrasound was repeated at 24 months (window 18 to 30 months). All patients provided written informed consent. The ethical committee of the Slotervaart Hospital and Reade (Amsterdam, the Netherlands) and the boards of directors at each hospital approved the trial protocol before local conduct started.

### Data Collection

Data were collected by the central study coordinators (SH and MG) in electronic case report forms using Castor EDC [42]. Preoperatively, data on demographics, presence of comorbidity, and preoperative medication use were collected. Hypertension, type 2 diabetes, and pain syndrome were defined as earlier described [30]. Dyslipidemia was defined as the presence of at least one of the following: known dyslipidemia, use of lipid-lowering drugs, a high-density lipoprotein cholesterol ≤ 0.9 mmol/L, low-density lipoprotein cholesterol ≥ 5.0 mmol/L, total cholesterol ≥ 6.5 mmol/L, or triglycerides ≥ 5.0 mmol/L. The individual potency of each statin was determined according to the meta-analysis by Law et al. [30, 43]. Ethnicity was self-reported by the trial participants. Categories consisted of Dutch, the four major non-Western ethnic groups in the Netherlands (Surinamese, Dutch Caribbean, Moroccan, Turkish), a category for all other ethnic groups, and a category for individuals with multiethnic backgrounds. Postoperatively, information on weight loss was gathered at 6, 12, and 24 months. Percent total weight loss (%TWL) was calculated by the following formula: %TWL = [(initial weight – postoperative weight)]/initial weight] × 100.

### Study Outcomes

The primary endpoint was the occurrence of symptomatic gallstone disease, defined as hospital admission or hospital visit for symptomatic gallstone disease within 24 months after bariatric surgery. Symptomatic gallstone disease comprised acute biliary pancreatitis, acute cholecystitis, choledocholithiasis, cholangitis, and biliary colics. Each different etiology of symptomatic gallstone disease was diagnosed based on a specific combination of predefined clinical characteristics, laboratory results, and imaging findings. The definitions are described in detail in the published trial protocol [40] Primary endpoint adjudication was done by a blinded adjudication committee. In patients without gallstones before surgery, we assessed the formation of gallstones and/or sludge by ultrasound (window 18 to 30 months). The group of patients with gallstones and/or sludge on the follow-up ultrasound consisted of symptomatic and asymptomatic patients.

### Statistical Analysis

Logistic regression analysis was used to evaluate associations between patient characteristics and (1) the occurrence of symptomatic gallstone disease and (2) the formation of gallstones and/or sludge after bariatric surgery. Variables with known clinical importance and/or with a *p*-value of 0.10 or less in the univariable analysis were selected for multivariable analysis. Stepwise backward elimination was used to derive a final model: In each subsequent step, the least significant variable in the model was removed until all remaining variables had individual *p*-values smaller than 0.10. To take into account the effect of the intervention of the UPGRADE trial, three different strategies were conducted. First, the intervention was included in both multivariable models. Second, both analyses were repeated in patients assigned to placebo. Third, interactions between the intervention and patient characteristics were explored. In case of a significant interaction, the interaction term was included in the multivariable model. All statistical tests were two-sided and a p-value of less than 0.05 was considered significant. Statistical analyses were performed using SPSS statistics for Windows (version 26, Armonk, NY: IBM Corp).

## Results

### Study Population

**Fig. 1:**
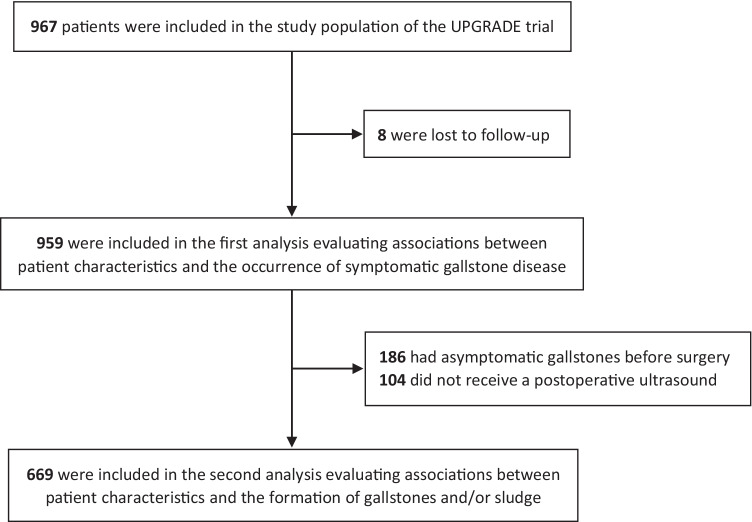
Flow diagram

The study population of the UPGRADE trial consisted of 967 patients, of whom 959 were available for primary endpoint assessment at 24 months (Fig. 1). Demographic and clinical characteristics are shown in Table 1. The mean age (± standard deviation) was 45.1 ± 11.1 years, and most patients were female (766 [80%]) and had a Dutch background (734 [77%]). The mean weight and body mass index at surgery were 116.0 ± 18.0 kg and 40.2 ± 4.7 kg/m^2^, respectively. Hypertension was the most common comorbidity (468 [49%]), and about one-fifth (186 [19%]) of the patients was diagnosed with asymptomatic gallstones before surgery. The majority received a laparoscopic RYGB (881 [92%]), and half of the patients was assigned to receive UDCA. Mean %TWL was 23.0 ± 5.4 at 6 months, 29.4 ± 7.4 at 12 months, and 29.4 ± 8.9 at 24 months. A subset of the study population—consisting of 669 patients without gallstones before surgery who received a postoperative ultrasound—shared all other characteristics (Table 1).

**Table 1:** Demographic and clinical characteristics of the total study population and a subset consisting of patients without gallstones before surgery who received a postoperative ultrasound

	Study population (*n* = 959)	Subset (*n* = 669)
Preoperative		
Age – years	45.1 ± 11.1	44.8 ± 11.3
Female gender – *n* (%)	766 (80)	519 (78)
Ethnicity – *n* (%)		
- Dutch	734 (77)	499 (75)
- Surinamese	55 (6)	45 (7)
- Dutch Caribbean	28 (3)	21 (3)
- Moroccan	18 (2)	14 (2)
- Turkish	16 (2)	11 (2)
- Other	49 (5)	40 (6)
- Multiple	59 (6)	39 (6)
Weight at surgery – kg	116.0 ± 18.0	115.7 ± 18.3
Body mass index at surgery – kg/m^2^	40.2 ± 4.7	40.1 ± 4.8
Hypertension – *n* (%)	468 (49)	311 (46)
Dyslipidemia – *n* (%)	301 (31)	208 (31)
Type 2 diabetes – *n* (%)	156 (16)	111 (17)
Pain syndrome – *n* (%)	81 (8)	61 (9)
Asymptomatic gallstones before surgery – *n* (%)	186 (19)	NA
Type of surgery – *n* (%)		
- RYGB	881 (92)	611 (91)
- Sleeve gastrectomy	78 (8)	58 (9)
Statin use – *n* (%)	164 (17)	122 (18)
- Low intensity	26 (3)	21 (3)
- Moderate intensity	85 (9)	64 (10)
- High intensity	53 (6)	37 (6)
Postoperative		
Assigned to UDCA – *n* (%)	475 (50)	331 (50)
%TWL at 6 months* – percent	23.0 ± 5.4	23.0 ± 5.4
%TWL at 12 months* – percent	29.4 ± 7.4	29.4 ± 7.5
%TWL at 24 months* – percent	29.4 ± 8.9	29.4 ± 9.2

Plus-minus values are means ± standard deviations. *N*, number; *RYGB*, Roux-en-Y gastric bypass; *UDCA*, ursodeoxycholic acid; *TWL*, total weight loss. *In the study population, data on weight was missing in 9 patients at 6 months, in 8 patients at 12 months, and in 5 patients at 24 months. In the subset, data on weight was missing in 1 patient at 6 and 12 months

### Factors Associated with Symptomatic Gallstone Disease

During 24 months of follow-up, 78 patients (8%) developed symptomatic gallstone disease after a median [IQR] of 316 [184 to 477] days. In patients with asymptomatic gallstones before surgery, 27 (15%) of 186 patients developed symptomatic gallstone disease. The results of the univariable and multivariable logistic regression analyses for the outcome occurrence of symptomatic gallstone disease are shown in Table 2. The presence of asymptomatic gallstones before surgery (odds ratio [OR] 2.40; 95% confidence interval [CI], 1.46 to 3.95; *p* < 0.001) and a higher %TWL at 24 months (OR 1.03; 95% CI 1.00 to 1.06; *p* = 0.04) were significantly associated with an increased risk for symptomatic gallstone disease after bariatric surgery in the univariable analysis. Advanced age (OR 0.96; 95% CI, 0.94 to 0.98; *p* < 0.001) and the use of statins (OR 0.38; 95% CI, 0.16 to 0.89; *p* = 0.03) were both significantly associated with a reduced risk. No dose–effect relationship was observed between the intensity of the statins and the occurrence of symptomatic gallstone disease (*p* = 0.32). In the multivariable analysis, the presence of a pain syndrome (OR 2.07; 95% CI 1.03 to 4.17; *p* = 0.04) and the presence of asymptomatic gallstones before surgery (OR 3.15; 95% CI 1.87 to 5.33, *p* < 0.001) were significantly associated with an increased risk. Advanced age (OR 0.95; 95% CI 0.93 to 0.97; *p* < 0.001) was protective for symptomatic gallstone disease after bariatric surgery, and UDCA prophylaxis did not reach statistical significance (OR 0.64; 95% CI 0.39 to 1.03; *p* = 0.07). Similar associations were observed in patients assigned to placebo (Supplementary Table 1). A significant interaction between the intervention (UDCA/placebo) and the presence of asymptomatic gallstones before surgery was identified in the univariable analysis (OR 2.83; 95% CI 1.02 to 7.86; *p* = 0.046). The final model of the multivariable analysis including this interaction is shown in Supplementary Table 2. Age and the presence of a pain syndrome were similarly associated with the risk for symptomatic gallstone disease after bariatric surgery as compared to the multivariable model without the interaction term. The effect of UDCA prophylaxis was significant in patients without gallstones before surgery (OR 0.46; 95% CI 0.25–0.85; *p* = 0.01).

**Table 2:** Association between patient characteristics and symptomatic gallstone disease in the study population (*n* = 959)

	Univariable		Multivariable†	
	OR (95% CI)	*p*-value	OR (95% CI)	*p*-value
Preoperative				
Age – years	0.96 (0.94 to 0.98)	< 0.001	0.95 (0.93 to 0.97)	< 0.001
Gender – female vs male	1.78 (0.90 to 3.53)	0.10	-	
Weight at surgery – kg	1.00 (0.98 to 1.01)	0.59	x*	
Body mass index at surgery – kg/m^2^	1.00 (0.95 to 1.05)	0.92	x*	
Hypertension – yes vs. no	0.63 (0.39 to 1.02)	0.06	-	
Dyslipidemia – yes vs. no	0.59 (0.34 to 1.02)	0.06	-	
Type 2 diabetes – yes vs. no	0.48 (0.22 to 1.07)	0.07	-	
Pain syndrome – yes vs. no	1.90 (0.96 to 3.76)	0.06	2.07 (1.03 to 4.17)	0.04
Asymptomatic gallstones before surgery** –** yes vs. no	2.40 (1.46 to 3.95)	< 0.001	3.15 (1.87 to 5.33)	< 0.001
Type of surgery – RYGB vs. sleeve gastrectomy	0.65 (0.31 to 1.36)	0.25	-	
Statin use – yes vs. no	0.38 (0.16 to 0.89)	0.03	-	
Postoperative				
Intervention – UDCA vs. placebo	0.65 (0.40 to 1.04)	0.07	0.64 (0.39 to 1.03)	0.07
%TWL at 6 months – percent	1.04 (0.99 to 1.08)	0.10	-	
%TWL at 12 months – percent	1.03 (1.00 to 1.06)	0.09	x*	
%TWL at 24 months – percent	1.03 (1.00 to 1.06)	0.04	x*	

*OR*, odds ratio; *CI*, confidence interval; *RYGB*, Roux-en-Y gastric bypass; *UDCA*, ursodeoxycholic acid; *TWL*, total weight loss. *Variables not included in the multivariable model. †Variables excluded from the model following stepwise backward elimination: gender, hypertension, dyslipidemia, type 2 diabetes, type of surgery, statin use, and %TWL at 6 months

### Factors Associated with the Formation of Gallstones and/or Sludge

Gallstones and/or sludge were observed on the follow-up ultrasound in 107 (16%) of the 669 patients without gallstones before surgery, of whom 56 (52%) were asymptomatic. The results of the univariable and multivariable logistic regression analyses for the outcome formation of gallstones and/or sludge are shown in Table 3. None of the patient characteristics was significantly associated with an increased risk for the formation of gallstones and/or sludge, although type 2 diabetes almost reached significance in the multivariable analysis (OR 1.80; 95% CI 0.97 to 3.34; *p* = 0.06). In both the univariable and multivariable analyses, advanced age (OR 0.98; 95% CI 0.96 to 1.00; *p* = 0.04), the use of statins (OR 0.42; 95% CI 0.20 to 0.90; *p* = 0.03), and UDCA prophylaxis (OR 0.47; 95% CI 0.30 to 0.73; *p* < 0.001) were significantly associated with a reduced risk. No dose–effect relationship was observed between the intensity of the statins and the formation of gallstones and/or sludge (*p* = 0.13). In patients assigned to placebo, only the use of statins was significantly associated with the formation of gallstones and/or sludge in the multivariable analysis (Supplementary Table 3). A significant interaction between the intervention and the presence of type 2 diabetes was identified in the univariable analysis (OR 3.79; 95% CI 1.27 to 11.31; *p* = 0.02). The final model of the multivariable analysis including this interaction is shown in Supplementary Table 4. Age and statin use were similarly associated with the formation of gallstones and/or sludge as compared to the multivariable model without the interaction term. The protective effect of UDCA prophylaxis was stronger in patients without type 2 diabetes compared to the entire subset (OR 0.37; 95% CI 0.23–0.61; *p* < 0.001).

**Table 3:** Association between patient characteristics and the formation of gallstones and/or sludge in the subset (*n* = 669)

	Univariable		Multivariable†	
	OR (95% CI)	*p*-value	OR (95% CI)	*p*-value
Preoperative				
Age – years	0.97 (0.96 to 0.99)	0.006	0.98 (0.96 to 1.00)	0.04
Gender – female vs male	1.14 (0.69 to 1.89)	0.61	-	
Weight at surgery – kg	1.00 (0.99 to 1.01)	0.60	x*	
Body mass index at surgery – kg/m^2^	1.02 (0.97 to 1.06)	0.49	x*	
Hypertension – yes vs. no	0.81 (0.53 to 1.23)	0.32	-	
Dyslipidemia – yes vs. no	0.67 (0.42 to 1.08)	0.10	-	
Type 2 diabetes – yes vs. no	1.19 (0.70 to 2.03)	0.52	1.80 (0.97 to 3.34)	0.06
Pain syndrome – yes vs. no	1.48 (0.77 to 2.84)	0.24	-	
Type of surgery – RYGB vs. sleeve gastrectomy	0.80 (0.40 to 1.59)	0.52	-	
Statin use – yes, no (%)	0.47 (0.24 to 0.90)	0.02	0.42 (0.20 to 0.90)	0.03
Postoperative				
Intervention – UDCA vs. placebo	0.46 (0.30 to 0.71)	< 0.001	0.47 (0.30 to 0.73)	< 0.001
%TWL at 6 months – percent	1.00 (0.97 to 1.04)	0.85	-	
%TWL at 12 months – percent	1.00 (0.97 to 1.03)	0.90	x*	
%TWL at 24 months – percent	1.01 (0.99 to 1.03)	0.42	x*	

*OR*, odds ratio; *CI*, confidence interval; *RYGB*, Roux-en-Y gastric bypass; *UDCA*, ursodeoxycholic acid; *TWL*, total weight loss. *Variables not included in the multivariable model. †Variables excluded from the model following stepwise backward elimination: gender, hypertension, dyslipidemia, pain syndrome, type of surgery, and %TWL at 6 months

## Discussion

In this prospective cohort study, we investigated associations between patient characteristics and the occurrence of symptomatic gallstone disease, and the formation of gallstones and/or sludge in patients who underwent bariatric surgery. Risk factors for symptomatic gallstone disease after bariatric surgery were a pain syndrome and the presence of asymptomatic gallstones before surgery, whereas advanced age was a protective factor. Besides UDCA prophylaxis, advanced age and the use of statins were found to reduce the formation of gallstones and/or sludge.

Obesity is a well-established risk factor for cholesterol gallstone disease. Consequently, asymptomatic gallstones are frequently encountered in patients scheduled for bariatric surgery. In this study, 186 (19%) patients had asymptomatic gallstones before surgery. Patients and treating physicians were both blinded for the preoperative ultrasound result, and therefore not aware of the presence of gallstones. Still, patients with asymptomatic gallstones before surgery were more likely to develop symptomatic gallstone disease afterwards than those without. Three retrospective cohort studies also included patients with asymptomatic gallstones and investigated their association with biliary complications or cholecystectomy after bariatric surgery [18, 26, 27]. In contrast to our finding, two of them did not observe a significant association [18, 27], while Chang et al. only identified a significant association in the univariable analysis [26]. The variation in study design, study population, and/or method for statistical analysis might explain the difference. Moreover, theoretically it also makes sense that patients with asymptomatic gallstones before surgery have an increased risk of developing symptomatic gallstones disease after bariatric surgery. First, they are at risk for the progression of asymptomatic to symptomatic disease, regardless of the bariatric procedure. Second, the bariatric procedure might foster the formation of new gallstones and/or sludge in already susceptible patients. Third, since abdominal complaints are common postoperatively and abdominal ultrasounds are frequently performed, there is a risk that misclassification may also contribute. Gallstones are by definition present in patients who had asymptomatic gallstones before surgery and therefore can be regarded as potential cause of symptoms. Among patients without gallstones before surgery, this can only be the case in those who develop new stones. Howbeit, no preventive measures are currently available for patients with asymptomatic gallstones before surgery. Prophylactic cholecystectomy is not warranted, because the majority of patients remains asymptomatic afterwards [7]. Likewise, UDCA prophylaxis does not seem to be indicated, since we have shown that it did not reduce the occurrence of symptomatic gallstone disease in these patients [41].

The presence of a pain syndrome was also identified as risk factor for the occurrence of symptomatic gallstone disease after bariatric surgery, which is in line with our previous finding that a pain syndrome increases the chance of undergoing cholecystectomy after bariatric surgery [30] In our previous paper, we speculated that patients with a pain syndrome are more likely to visit a doctor in case they experience abdominal pain, often resulting in imaging of the abdomen. We all know that postoperatively, the probability to find gallstones during imaging is high. Hence, a very thorough diagnostic workup of abdominal complaints after bariatric surgery should be done in order to accurately diagnose symptomatic gallstones disease to prevent unnecessary cholecystectomies. Accordingly, we found no association between the presence of a pain syndrome and the formation of gallstones and/or sludge, since patients received the postoperative ultrasound for study purposes rather than complaints.

In the present study, we did not find a significant independent association between %TWL and symptomatic gallstone disease, nor between %TWL and the formation of gallstones and/or sludge. At first, this may seem contradictory since rapid weight loss is a well-established risk factor for cholesterol gallstone disease, and the reason why bariatric patients are at risk. However, the fact that all bariatric patients lose weight might explain why we did not find an association in our cohort. A thorough literature search yielded inconsistent findings. Like our study, many studies did not find a significant association between weight loss and gallstone disease in a bariatric population [2–4, 10, 12, 14, 16–19, 22–26, 28, 29, 32, 33, 37–39], whereas several others did [8, 9, 11, 13, 15, 21, 27, 30, 35, 36, 44]. Hence, it seems unlikely that %TWL (or another weight loss outcome measure) can be of help to identify specific bariatric patients at risk and to guide preventive treatment in the near future.

In the general population, metabolic abnormalities such as dyslipidemia, type 2 diabetes, and insulin resistance predispose patients to cholesterol gallstone disease [1]. In our study, dyslipidemia and type 2 diabetes were not significantly associated with our study outcomes. Hypothetically, it is unlikely that the presence of preoperative comorbidities could play a major role in the prediction of which bariatric patient is at high risk, since their existence is rapidly decreased after the bariatric procedure.

In line with several other studies who have shown that advanced age decreases the risk on cholecystectomy after bariatric surgery [30, 31, 36, 37], we have found that older patients have a decreased risk on symptomatic gallstone disease and gallstone/sludge formation.

Apart from UDCA prophylaxis as being protective in patients without gallstones before surgery, lipid-lowering drugs might also be able to reduce gallstone disease and gallstone formation after bariatric surgery. Our univariable analyses showed that preoperative statin use significantly reduced the occurrence of symptomatic gallstone disease and the formation of gallstones and/or sludge after bariatric surgery. However, using stepwise backward elimination, statin use was only maintained in the multivariable model for the formation of gallstones and/or sludge. In contrast to our previous retrospective study, we did not observe a dose–effect relationship [30]. Yet, these results should be confirmed in a prospective intervention study.

Some studies have reported that in the general population, the prevalence of gallstone disease varies by ethnic group as the result of different genetic susceptibility and environmental factors [1]. Therefore, ethnicity might be associated with our outcomes. However, the non-Dutch ethnic groups were too small to properly evaluate this association. Moreover, an etiological principle why ethnicity would differentially affect the risk of gallstones in an obese population during rapid weight loss seems lacking.

Strengths of this cohort study include the prospective data collection, blinded endpoint adjudication, and the low number of losses to follow-up in the UPGRADE trial, which resulted in a valid and complete data set. On the other hand, the use of randomized trial data might have introduced some selection bias because of the entry criteria, and the willingness to participate [45]. However, we have no indications that we have selected healthier participants, since the exclusion criteria were not extensive.

## Conclusion

Our findings suggest that young patients with a preoperative pain syndrome and/or asymptomatic gallstones before surgery are at increased risk for symptomatic gallstone disease after bariatric surgery. A prospective study to explore whether statins or other lipid-lowering drugs, either alone or in combination with UDCA prophylaxis, further reduce the burden of gallstones after bariatric surgery is desirable. Following this study, the development of an useful clinical prediction model will be a step forward in a tailored approach for the prevention of symptomatic gallstone disease and gallstone formation after bariatric surgery.

## Supplementary Information

Below is the link to the electronic supplementary material.

Supplementary file1 (DOCX 26 KB)
