# Supplementary material for: Risk Factors for Symptomatic Gallstone Disease and Gallstone Formation After Bariatric Surgery
Source: Obes Surg. 2022 Feb 10;32(4):1270–8. doi: 10.1007/s11695-022-05947-8 (PMC8933359; doi:10.1007/s11695-022-05947-8)
Supplement: Supplementary file 1 — Supplementary file1 (DOCX 26 KB) [file 11695_2022_5947_MOESM1_ESM.docx]

| **Supplementary Table 1. Association between patient characteristics and symptomatic gallstone disease in patients who were assigned to placebo (n=484)** | | | | |
| --- | --- | --- | --- | --- |
| **PREOPERATIVE** | **Univariable** | | **Multivariable†** | |
|  | **OR (95% CI)** | **p-value** | **OR (95% CI)** | **p-value** |
| **Age** – years | 0.95 (0.93 to 0.98) | <0.001 | 0.94 (0.92 to 0.97) | <0.001 |
| **Gender** – female vs male | 1.50 (0.65 to 3.47) | 0.34 | - |  |
| **Weight at surgery** – kg | 1.00 (0.98 to 1.02) | 0.85 | x* |  |
| **Body mass index at surgery** – kg/m^2^ | 1.01 (0.95 to 1.08) | 0.73 | x* |  |
| **Hypertension** – yes vs no | 0.48 (0.25 to 0.90) | 0.02 | - |  |
| **Dyslipidemia** – yes vs no | 0.44 (0.21 to 0.94) | 0.03 | - |  |
| **Type 2 diabetes** – yes vs no | 0.26 (0.08 to 0.87) | 0.03 | - |  |
| **Pain syndrome** – yes vs no | 2.51 (1.08 to 5.81) | 0.03 | 3.00 (1.25 to 7.19) | 0.01 |
| **Asymptomatic gallstones before surgery –** yes vs no | 1.53 (0.76 to 3.08) | 0.23 | 2.23 (1.06 to 4.70) | 0.03 |
| **Type of surgery** – RYGB vs sleeve gastrectomy | 0.97 (0.33 to 2.84) | 0.95 | - |  |
| **Statin use** – yes vs no | 0.28 (0.08 to 0.92) | 0.04 | - |  |
| **POSTOPERATIVE** | | | | |
| **%TWL at 6 months** – per | 1.04 (0.99 to 1.10) | 0.11 | - |  |
| **%TWL at 12 months** – percent | 1.04 (1.00 to 1.08) | 0.07 | x* |  |
| **%TWL at 24 months** – percent | 1.03 (1.00 to 1.07) | 0.06 | x* |  |
| OR: odds ratio; CI: confidence interval; RYGB: Roux-en-Y gastric bypass; TWL: total weight loss. *Variables not included in the multivariable model. †Variables excluded from the model following stepwise backward elimination: gender, hypertension, dyslipidemia, type 2 diabetes, type of surgery, statin use, and %TWL at 6 months. | | | | |

| **Supplementary Table 2. Multivariable association between patient characteristics and symptomatic gallstone disease in the study population; significant interaction term included (n=959)** | | |
| --- | --- | --- |
| **PREOPERATIVE** | **Final model*** | |
|  | **OR (95% CI)** | **p-value** |
| **Age** – years | 0.95 (0.93 to 0.97) | <0.001 |
| **Pain syndrome** – yes vs no | 2.02 (1.00 to 4.08) | 0.05 |
| **Asymptomatic gallstones before surgery –** yes vs no | 2.06 (1.00 to 4.25) | 0.05 |
| **Intervention** – UDCA vs placebo | 0.46 (0.25 to 0.85) | 0.01 |
| **Interaction**† | 2.56 (0.91 to 7.24) | 0.08 |
| OR: odds ratio; CI: confidence interval. *Variables not included in the multivariable model: weight at surgery, body mass index at surgery, % total weight loss at 12 and 24 months; variables excluded from the model following stepwise backward elimination: gender, hypertension, dyslipidemia, type 2 diabetes, type of surgery, statin use, and % total weight loss at 6 months. †Interaction: intervention x asymptomatic gallstones before surgery. | | |

| **Supplementary Table 3. Association between patient characteristics and the formation of gallstones and/or sludge in patients without gallstones before surgery assigned to placebo (n=338)** | | | | |
| --- | --- | --- | --- | --- |
| **PREOPERATIVE** | **Unadjusted** | | **Adjusted†** | |
|  | **OR (95% CI)** | **p-value** | **OR (95% CI)** | **p-value** |
| **Age** – years | 0.97 (0.95 to 1.00) | 0.03 | - |  |
| **Sex** – female vs male | 0.90 (0.49 to 1.67) | 0.74 | - |  |
| **Weight at surgery** – kg | 1.01 (1.00 to 1.03) | 0.20 | x* |  |
| **Body mass index at surgery** – kg/m^2^ | 1.03 (0.98 to 1.09) | 0.21 | x* |  |
| **Hypertension** – yes vs no | 0.70 (0.41 to 1.19) | 0.18 | - |  |
| **Dyslipidemia** – yes vs no | 0.55 (0.30 to 1.00) | 0.05 | - |  |
| **Type 2 diabetes** – yes vs no | 0.68 (0.33 to 1.37) | 0.28 | - |  |
| **Pain syndrome** – yes vs no | 1.41 (0.62 to 3.16) | 0.41 | - |  |
| **Type of surgery** – RYGB vs sleeve gastrectomy | 0.77 (0.28 to 2.09) | 0.60 | - |  |
| **Statin use** – yes vs no | 0.39 (0.17 to 0.89) | 0.02 | 0.39 (0.17 to 0.89) | 0.02 |
| **POSTOPERATIVE** | | | | |
| **%TWL at 6 months** – percent | 1.00 (0.95 to 1.05) | 0.92 | - |  |
| **%TWL at 12 months** – percent | 1.00 (0.96 to 1.02) | 0.53 | x* |  |
| **%TWL at 24 months** – percent | 1.00 (0.97 to 1.03) | 0.98 | x* |  |
| OR: odds ratio; CI: confidence interval; RYGB: Roux-en-Y gastric bypass; TWL: total weight loss. *Variables not included in the multivariable model. †Variables excluded from the model following stepwise backward elimination: gender, hypertension, dyslipidemia, type 2 diabetes, type of surgery, statin use, and %TWL at 6 months. | | | | |

| **Supplementary Table 4. Multivariable association between patient characteristics and the formation of gallstones and/or sludge in patients without gallstones before surgery; significant interaction term included (n=669)** | | |
| --- | --- | --- |
| **PREOPERATIVE** | **Final model*** | |
|  | **OR (95% CI)** | **p-value** |
| **Age** – years | 0.98 (0.96 to 1.00) | 0.046 |
| **Type 2 diabetes** – yes vs no | 1.11 (0.51 to 2.38) | 0.80 |
| **Statin use** – yes vs no | 0.42 (0.20 to 0.90) | 0.03 |
| **Intervention** – UDCA vs placebo | 0.37 (0.23 to 0.61) | <0.001 |
| **Interaction**† | 3.89 (1.27 to 11.90) | 0.02 |
| OR: odds ratio; CI: confidence interval. *Variables not included in the multivariable model: weight at surgery, body mass index at surgery, % total weight loss at 12 and 24 months; variables excluded from the model following stepwise backward elimination: gender, hypertension, dyslipidemia, type 2 diabetes, type of surgery, statin use, and % total weight loss at 6 months. †Interaction: intervention x type 2 diabetes. | | |
